# Direct and indirect genetic effects on triglycerides through omics and correlated phenotypes

**DOI:** 10.1186/s12919-018-0118-9

**Published:** 2018-09-17

**Authors:** Anne E. Justice, Annie Green Howard, Lindsay Fernández-Rhodes, Misa Graff, Ran Tao, Kari E. North

**Affiliations:** 1 Department of Epidemiology, University of North Carolina, Chapel Hill, NC USA; 2 Biomedical and Translational Informatics, Geisinger Health, Danville, PA USA; 3 Department of Biostatistics, University of North Carolina, Chapel Hill, NC USA; 4 Carolina Population Center, University of North Carolina, Chapel Hill, NC USA; 5 Department of Biostatistics, Vanderbilt University Medical Center, Nashville, TN USA

## Abstract

Even though there has been great success in identifying lipid-associated single-nucleotide polymorphisms (SNPs), the mechanisms through which the SNPs act on each trait are poorly understood. The emergence of large, complex biological data sets in well-characterized cohort studies offers an opportunity to investigate the genetic effects on trait variability as a way of informing the causal genes and biochemical pathways that are involved in lipoprotein metabolism. However, methods for simultaneously analyzing multiple omics, environmental exposures, and longitudinally measured, correlated phenotypes are lacking. The purpose of our study was to demonstrate the utility of the structural equation modeling (SEM) approach to inform our understanding of the pathways by which genetic variants lead to disease risk. With the SEM method, we examine multiple pathways directly and indirectly through previously identified triglyceride (TG)-associated SNPs, methylation, and high-density lipoprotein (HDL), including sex, age, and smoking behavior, while adding in biologically plausible direct and indirect pathways. We observed significant SNP effects (*P* < 0.05 and directionally consistent) on TGs at visit 4 (TG4) for five loci, including rs645040 (*DOCK7*), rs964184 (*ZPR1*/*ZNF259*), rs4765127 (*ZNF664*), rs1121980 (*FTO*), and rs10401969 (*SUGP1*). Across these loci, we identify three with strong evidence of an indirect genetic effect on TG4 through HDL, one with evidence of pleiotropic effect on HDL and TG4, and one variant that acts on TG4 indirectly through a nearby methylation site. Such information can be used to prioritize candidate genes in regions of interest, inform mechanisms of action of methylation effects, and highlight possible genes with pleiotropic effects.

## Background

Lipid traits, such as triglyceride (TG) and high-density lipoprotein (HDL) cholesterol concentrations, are highly heritable; estimates range from 20 to > 70%, with common variants estimated to explain approximately 30 to 33% of the variance for these traits [1, 2]. Genome-wide association studies (GWAS) have identified more than 100 SNPs associated with lipid traits, many of which are shared across more than one lipid trait [1–8]. Even though there has been great success in identifying lipid-associated SNPs, the mechanisms through which these SNPs act on each trait are poorly understood. The emergence of large, complex biological data sets in well-characterized cohort studies offers an opportunity to investigate the genetic effects on trait variability as a way of informing the causal genes and biochemical pathways that are involved in lipoprotein metabolism. However, methods for simultaneously analyzing multiple omics, environmental exposures, and longitudinally measured, correlated phenotypes are lacking.

The purpose of our study was to demonstrate the utility of the structural equation modeling (SEM) approach to inform our understanding of the pathways by which genetic variants lead to disease risk. With the SEM method, we can examine multiple pathways directly and indirectly through previously identified TG-associated SNPs, methylation, and HDL, including sex, age, and smoking behavior, while adding in biologically plausible direct and indirect pathways. Although SEM has been used to examine the influence of genetic variants on disease through environmental exposures [9], on gene expression [10], and pleiotropy [11], to our knowledge this will be the first study to investigate pathways between GWAS-established SNPs and a disease risk factor while accounting for environmental exposures, correlated phenotypes, and epigenetic markers simultaneously using an SEM framework. Thus, using the GAW20 data, we will show the usefulness of the SEM results to inform the prioritization of candidate genes in regions of association, inform mechanisms of action of methylation effects, and inform possible genes with pleiotropic effects.

## Methods

### Study sample

GAW20data were provided by the Genetics of Lipid Lowering Drugs and Diet Network (GOLDN) study. Individual genetic and phenotypic data are drawn from a total of 1105 adults from 188 families. Of these, 810 individuals from 172 families have been genotyped on the Affymetrix 6.0 (Affymetrix, Inc., Santa Clara, CA, USA).

### Phenotypes/covariates

Our analyses focused on fasting TG as the primary outcome measure. Secondary outcomes included DNA methylation and HDL. Of those with genotype data, 707 participants had whole-genome methylation data measured from CD4+ T cells at visit 2. The HM450K array was used to measure DNA methylation (Illumina, Inc., San Diego, CA, USA) following bisulphite conversion. The platform detects methylation status of 485,577 CpG (cystine-phosphate-guanine) sites by sequencing-based genotyping of bisulphite-treated DNA. The methylation score for each CpG is reported as a β value, ranging from 0 (nonmethylated) to 1 (completely methylated), according to the intensity ratio of detected methylation. We calculated principal components (PCs) using methylation β values across all CpGs in R (v3.3.1) and used the first four PCs to adjust to control for cell purity and batch effects prior to performing association analyses. Covariates included sex, baseline age, and study center. Additionally, we adjusted for baseline smoking status, as had been done in previous genetic and methylation association analyses [12–15].

### SNP and CpG selection

We selected established TG-associated SNPs [2, 16] available on the Affymetrix 6.0, array (Table 1) to evaluate direct and indirect SNP effects on TG in our SEM framework. To evaluate indirect effects through methylation, we searched for CpG sites near our tag variant (±10 kb) for inclusion; however, as the focus of this paper is direct and indirect SNP effects on TG at visit 4 (TG4), we do not report significant direct effects of CpG methylation on TG. For one SNP, rs10503669, there were no CpGs within 10 kb, so we extended the window to ±20 kb (Table 1). Table 1 provides the total number of CpGs available on the HM450K array that have passed quality control (QC) filters and are within ±10 kb of our tag SNPs.

**Table 1:** TG-associated SNPs and nearby CpGs included in SEM models

Rsid	Chr	Pos (hg19)	Gene	EA/OA	EAF	PMID	# of CpGs ± 10 kb	CpGs included in final model^a^	Parameters	# Variables (Dependent/Independent)
rs1748195	1	63,049,593	*DOCK7*	G/C	0.636	18193043	1	cg00161770	55	9/6
rs645040	3	135,926,622	*RPL31P23/PCCB*	A/C	0.807	24097068	2	cg15219878	55	9/6
rs998584	6	43,757,896	*VEGFA*	C/A	0.543	24097068	6	cg03143046, cg01353538, cg20940044, cg25373579, cg23879496, cg12682870	75	14/6
rs10503669	8	19,847,690	*LPL*	G/T	0.909	18193043	0	cg18449136^a^	55	9/6
rs964184	11	116,154,127	*ZPR1/ZNF259*	G/C	0.874	24097068	24	cg06595719, cg14815609, cg05862431, cg11835342, cg14371153, cg17490921	75	14/6
rs4765127	12	123,026,120	*ZNF664*	G/T	0.672	24097068	13	cg19078769, cg00201185, cg10922530, cg02647265	67	12/6
rs4775041	15	58,674,695	Intergenic	G/C	0.720	18193043	1	cg25188724	55	9/6
rs3198697	16	15,129,940	*PDXDC1*	C/T	0.569	24097068	5	cg16724811, cg06978461, cg03245889, cg03928410, cg26985681	71	13/6
rs1121980	16	53,809,247	*FTO*	C/T	0.529	24097068	2	cg02252501, cg03312170	59	10/6
rs8077889	17	41,878,166	*C17orf105/MPP3*	T/G	0.798	24097068	2	cg13317831, cg01571583	59	10/6
rs7248104	19	7,224,431	*INSR*	G/A	0.575	24097068	2	cg09779027, cg00428638	59	10/6
rs10401969	19	19,407,718	*SUGP1*	A/G	0.929	24097068	5	cg00477287, cg01313994, cg01559787, cg08112740, cg19643441	71	13/6

*Abbreviations: Chr* chromosome, *EA* effect allele, *OA* other allele, *PMID* pubmed article ID number, *Pos* base pair position on chromosome

^a^CpGs within 10 kb ± and included in the final model are listed for all SNPs except rs10503669, which had only one CpG < 20 kb ±

### SEM

**Fig. 1:**
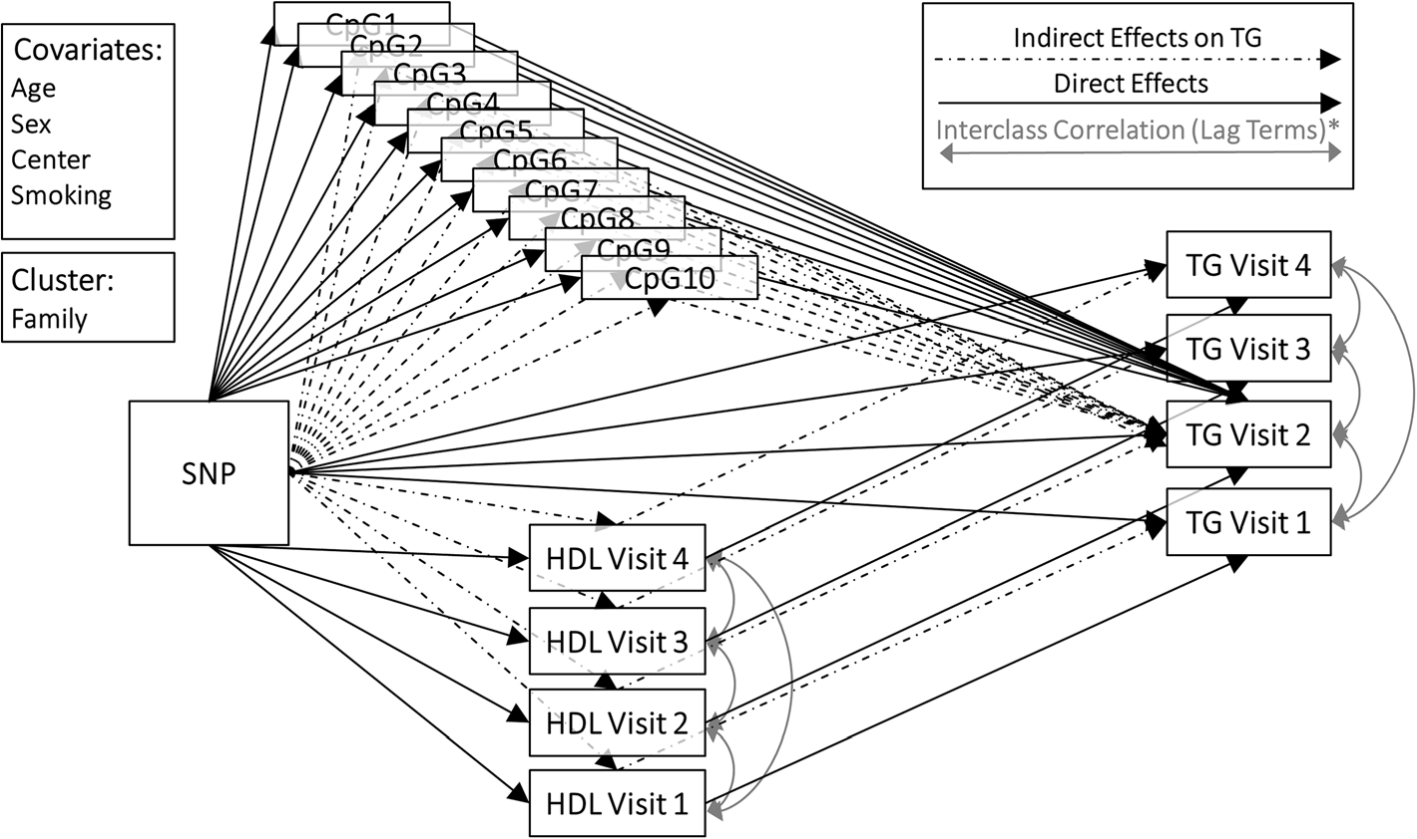
Diagram illustrating the full SEM model

For the SEM, we defined a structure a priori based on hypothesized pathways to evaluate how certain covariates directly and indirectly influenceTG4.Our SEM framework estimates all plausible pathways simultaneously, so each estimate can be considered conditional on all other possible pathways modeled within each locus. A lag effect for TG and HDL was included to allow current values to relate to the time point immediately prior, thereby allowing for covariates to have an indirect effect on TG and/or HDL through their impact on a previous time point. Huber-White sandwich estimation (HSE) was used to account for family relatedness and correlation within a household [17]. Root mean square error of approximation (RMSEA), comparative fit index (CFI), and Tucker-Lewis index (TLI) were examined to ensure appropriate model fit. As a consequence of the small sample size compared to model complexity we considered RMSEA values ≥0.10 and CFI or TLI ≤0.9 as an indicator of poor model fit [18–20]. For each locus, if our full model did not meet these fit criteria, nonsignificant CpGs (*P* > 0.2) were removed from the model and reevaluated for model fit. For all loci, model fit criteria were met following this step. Figure 1 illustrates the full models and shows all segments along pathways. Mplus Version 7.4 was used for all SEM; maximum likelihood estimation was used. Even though our SEM framework simultaneously estimated the effect of SNP across each segment of the modeled pathways, we report only direct and indirect effects on TG4 for the SNPs that reached nominal significance (*P* < 0.05) and displayed directionally consistent effects with previous GWAS findings.

## Results

Our sample included up to 707 participants of the GOLDN study (50% women); the average age of participants at the time of the baseline examination was 48 years. It is worth noting that all participants in the GOLDN study received treatment with fenofibrates between visits 2 and 3, which resulted in a decrease in mean TG and a decrease in the variance (mean[SD] TG1 = 106.35[106.35]; TG2 = 140.16[99.34]; TG3 = 92.26[57.41]; TG4 = 90.14[55.07]).

**Fig. 2:**
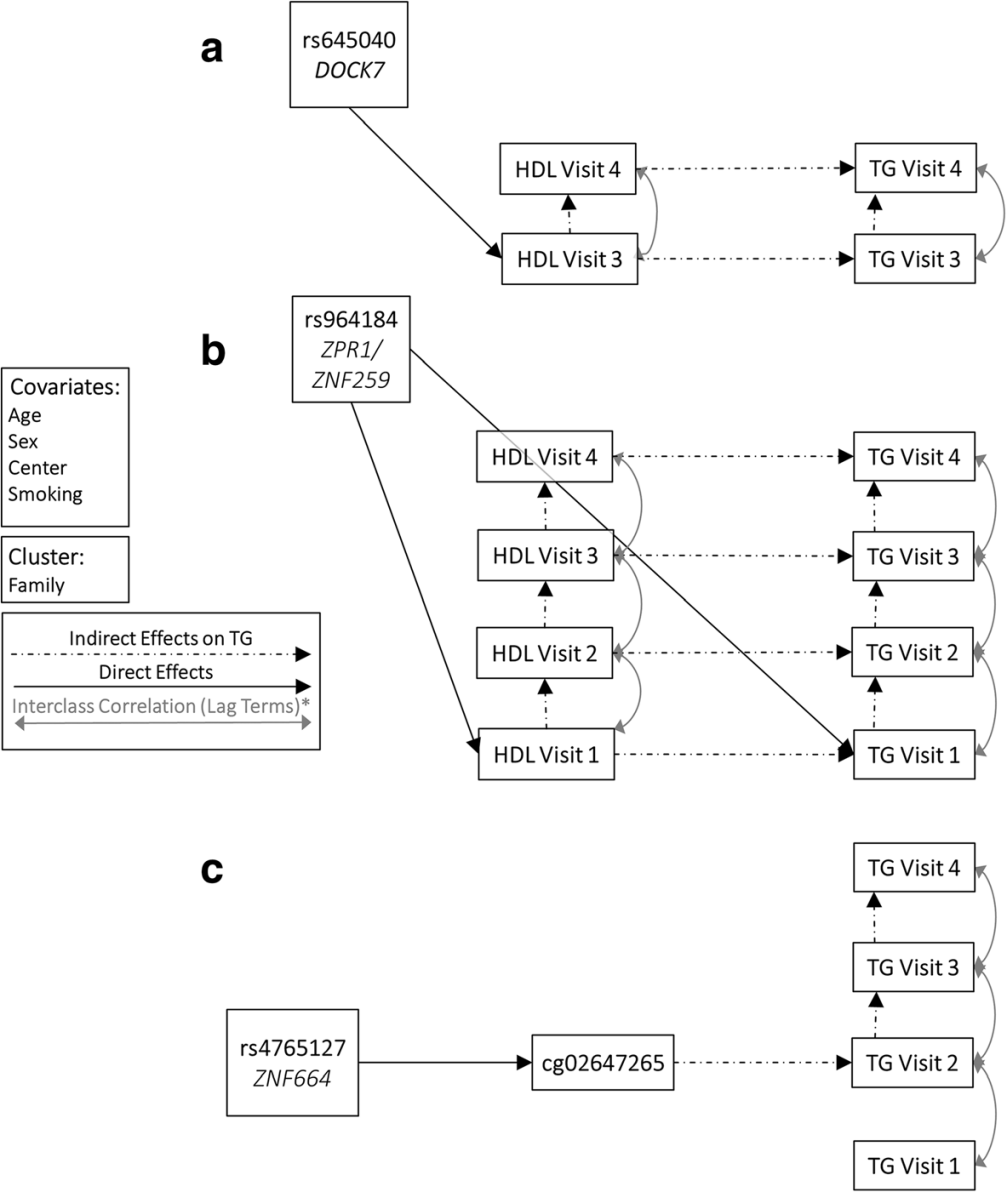
SEM models for SNPs with significant effects (*P* < 0.05) on TG4. **a** Diagram illustrating all nominally significant associations on TG at the rs645040 locus. **b** Diagram illustrating all nominally significant associations on TG at the rs964184 locus. **c** Diagram illustrating all nominally significant associations on TG at the rs4775041 locus

We observed significant indirect SNP effects (*P* < 0.05 and directionally consistent) on TG4 for five loci (Table 2), including rs645040 (*DOCK7*), rs964184 (*ZPR1*/*ZNF259*), rs4765127 (*ZNF664*), rs1121980 (*FTO*), andrs10401969 (*SUGP1*). We did not identify any significant direct effects of any SNP on TG4 after accounting for indirect effects through early measured TG, HDL or CpG methylation. For both rs645040 (Fig. 2a) and rs1121980, we observe a significant direct effect on HDL at visit three (HDL3) through which the SNP has a significant (*P* value < 0.05 and directionally consistent) indirect effect on TG4. Both variants were associated with TG in previous GWAS; rs1121980 was also associated with HDL [2]. Although rs645040 was not previously associated with HDL, rs483465, which lies only approximately 120 kb upstream of rs645040 (*R*^2^ = 0.904), has been associated with HDL. For both of these loci, the effect previously observed on TG and HDL does not appear to be a result of pleiotropy, but rather an indirect effect of the SNP on TG through its effects on HDL. Similarly, rs10401969, which displays a significant indirect effect through HDL at visit 1 (HDL1) on TG4, does not display a significant independent direct effect on TG. Even though this locus has not been associated with HDL, it has been associated with TG, low-density lipoprotein (LDL) cholesterol, and total cholesterol (TC).

**Table 2:** Parameter estimates for significant pathways from SNP, through intermediate exposure, to TG4

Intermediate	Pathways	Beta	SE	P	RMSEA	CFI	TLI
rs645040							
Through HDL3	SNP → HDL3 → HDL4 → TG4	0.229	0.115	0.046	0.083	0.96	0.93
	SNP → HDL3 → TG3 → TG4	0.597	0.286	0.037			
rs964184							
Through TG1	SNP → TG1 → TG2 → TG3 → TG4	−12.48	2.406	**< 0.001**	**0.07**	**0.94**	**0.92**
Through HDL1	SNP → HDL1 → HDL2 → TG2 → TG3 → TG4	−0.819	0.392	0.037			
	SNP → HDL1 → HDL2 → HDL3 → TG3 → TG4	−2.204	0.903	0.015			
	SNP → HDL1 → HDL2 → HDL3 → HDL4 → TG4	− 0.857	0.382	0.025			
	SNP → HDL1 → TG1 → TG2 → TG3 → TG4	−2.568	1.011	0.011			
rs4765127							
Through cg02647265	SNP → cg02647265 → TG2 → TG3 → TG4	0.323	0.153	0.034	0.074	0.95	0.92
rs1121980							
Through HDL3	SNP → HDL3 → TG3 → TG4	−0.547	0.249	0.028	0.08	0.96	0.93
rs10401969							
Through HDL1	SNP → HDL1 → TG1 → TG2 → TG3 → TG4	2.552	1.166	0.029	0.076	0.94	0.91
	SNP → HDL1 → HDL2 → HDL3 → TG3 → TG4	2.098	0.968	0.03			
	SNP → HDL1 → HDL2 → HDL3 → HDL4 → TG4	0.801	0.407	0.049			

We highlight all significant pathways with a focus only on significant SNP effects (*P* value < 0.05 and directionally consistent). Bonferroni significant pathways (*P* value < 0.004) are bolded

For rs964184 (*ZPR1*/*ZNF259*), which was previously associated with both HDL and TG [2], we identified direct effects on both HDL1 and TG1 through which the SNP displays a significant (*P* < 0.05 and directionally consistent) effect on TG4 (Fig. 2b). Also, the effect of this locus on TG across all visits remains significant after Bonferroni correction (*P* value < 0.004, 0.05/12 loci examined), as does the total effect of the SNP on TG4 accounting for all proposed pathways. Contrary to the three loci mentioned above, we do find evidence of an independent direct association for rs964184 with both TG and HDL. This suggests a model whereby a common single mechanism affects multiple lipoprotein concentrations, which is indicative of true pleiotropy. Indeed, the complexity of lipoprotein metabolism means that genes and biochemical pathways can be involved in metabolism of several lipoprotein classes [21].

Finally, we also observed a nominally significant (*P* value < 0.05) and directionally consistent indirect effect for rs4765127 (*ZNF664*) on TG4through a nearby CpG (cg02647265), which lies 4 kb upstream of our tag SNP in the 5′UTR (untranslated region) of *CCDC92*, the gene adjacent to *ZNF664*. While our tag SNP lies within *ZNF664*, variants within *CCDC92* also have been associated with multiple lipid levels and lipoprotein size, also suggesting *CCDC92* as a candidate gene for this region [22, 23]. Even though this variant was associated with both HDL and TG in previous GWAS [2], we found no evidence of a direct association of this SNP on either phenotype (Fig. 2c). Although we do observe an effect of the SNP mediated through a nearby CpG on TG, we did not explicitly model an association with the CpG on HDL. Because of the proximity of cg02647265 to the 5′ end of two genes (*CCDC92*and *ZNF664*), further investigation into the association of this CpG with expression is warranted to further elucidate the causal gene underlying this GWAS association signal.

## Discussion

We aimed to highlight the utility and flexibility of SEM for adding to our understanding of the genetic underpinnings of disease risk by investigating pathways between GWAS-established SNPs and a disease risk factor, TG, through correlated phenotypes and epigenetic markers simultaneously. There is substantial interest in the field for approaches to integrate multiple types of phenotypic and omics data so that a better understanding of disease mechanisms can be achieved. Using the GAW data, we were able to determine if the previously observed SNP effects on TG could be explained by an indirect effect of the SNP through HDL and nearby CpG sites. We identified three loci where associations with TG were indirect through HDL and one locus where the effects of SNPs on TG were mediated through methylation. Such information can be used to prioritize candidate genes in regions of association, inform mechanisms of action of methylation effects, and highlight possible genes with pleiotropic effects.

Although the examples highlighted herein demonstrate the utility and flexibility of SEM to inform mechanistic underpinnings of GWAS loci, our study is limited by the small set of variables available to investigate in the complex SEM. For example, only direct genotypes, methylation, HDL, and TG values were available. Lastly, it is also possible for nominally significant associations between CpGs and TG to be mediated through HDL, but because of model complexity and limited sample size, we did not test this explicitly.

## Conclusions

The majority of investigations into the genetic underpinnings of TG do not take advantage of the wealth of longitudinal data available in many large cohort studies. Additionally, there is a dearth of comprehensive studies that incorporate genetic, epigenetic, and correlated phenotypic data to investigate pathways through which genetic variants influence trait variance. To address this important research gap, we capitalized on extant genotypic data at known TG-associated loci, longitudinal assessments of TG and HDL, smoking exposure, and methylation available through the GAW20 to explore the utility and flexibility of the SEM framework for informing mechanistic insights at GWAS loci. In future investigations, the proposed approach can be easily extended to accommodate additional and longitudinal omics data, ultimately assisting researchers in better identifying the mechanist pathways through which genetic variants influence trait variance.

## References

[1] Aulchenko YS, Ripatti S, Lindqvist I, Boomsma D, Heid IM, Pramstaller PP, Penninx BW, Janssens AC, Wilson JF, Spector T (2009). Loci influencing lipid levels and coronary heart disease risk in 16 European population cohorts. Nat Genet.

[2] Willer CJ, Schmidt EM, Sengupta S, Peloso GM, Gustafsson S, Kanoni S, Ganna A, Chen J, Buchkovich ML, Mora S (2013). Global lipids genetics consortium: discovery and refinement of loci associated with lipid levels. Nat Genet.

[3] Comuzzie AG, Cole SA, Laston SL, Voruganti VS, Haack K, Gibbs RA, Butte NF (2012). Novel genetic loci identified for the pathophysiology of childhood obesity in the Hispanic population. PLoS One.

[4] Coram MA, Duan Q, Hoffmann TJ, Thornton T, Knowles JW, Johnson NA, Ochs-Balcom HM, Donlon TA, Martin LW, Eaton CB (2013). Genome-wide characterization of shared and distinct genetic components that influence blood lipid levels in ethnically diverse human populations. Am J Hum Genet.

[5] Ko A, Cantor RM, Weissglas-Volkov D, Nikkola E, Reddy PM, Sinsheimer JS, Pasaniuc B, Brown R, Alvarez M, Rodriguez A (2014). Amerindian-specific regions under positive selection harbour new lipid variants in Latinos. Nat Commun.

[6] Weissglas-Volkov D, Aguilar-Salinas CA, Nikkola E, Deere KA, Cruz-Bautista I, Arellano-Campos O, Muñoz-Hernandez LL, Gomez-Munguia L, Ordoñez-Sánchez ML, Reddy PM (2013). Genomic study in Mexicans identifies a new locus for triglycerides and refines European lipid loci. J Med Genet.

[7] Below JE, Parra EJ, Gamazon ER, Torres JM, Krithika S, Candille S, Lu Y, Manichakul A, Peralta-Romero J, Duan Q (2016). Meta-analysis of lipid-traits in Hispanics identifies novel loci, population-specific effects, and tissue-specific enrichment of eQTLs. Sci Rep.

[8] Teslovich TM, Musunuru K, Smith AV, Edmondson AC, Stylianou IM, Koseki M, Pirruccello JP, Ripatti S, Chasman DI, Willer CJ (2010). Biological, clinical and population relevance of 95 loci for blood lipids. Nature.

[9] Mi X, Eskridge KM, George V, Wang D (2011). Structural equation modeling of gene-environment interactions in coronary heart disease. Ann Hum Genet.

[10] Xiong M (2001). Structural equation models for pathway identification. Nat Genet.

[11] Li R, Tsaih SW, Shockley K, Stylianou IM, Wergedal J, Paigen B, Churchill GA (2006). Structural model analysis of multiple quantitative traits. PLoS Genet.

[12] Cole CB, Nikpay M, McPherson R (2015). Gene-environment interaction in dyslipidemia. Curr Opin Lipidol.

[13] Dumitrescu L, Carty CL, Taylor K, Schumacher FR, Hindorff LA, Ambite JL, Anderson G, Best LG, Brown-Gentry K, Bůžková P (2011). Genetic determinants of lipid traits in diverse populations from the population architecture using genomics and epidemiology (PAGE) study. PLoS Genet.

[14] Li X, Monda KL, Goring HH, Haack K, Cole SA, Diego VP, Almasy L, Laston S, Howard BV, Shara NM (2009). Genome-wide linkage scan for plasma high density lipoprotein cholesterol, apolipoprotein A-1 and triglyceride variation among American Indian populations: the strong heart family study. J Med Genet.

[15] Braun KVE, Dhana K, de Vries PS, Voortman T, van Meurs JBJ, Uitterlinden AG (2017). Epigenome-wide association study (EWAS) on lipids: the Rotterdam study. Clin Epigenetics.

[16] Willer CJ, Sanna S, Jackson AU, Scuteri A, Bonnycastle LL, Clarke R, Heath SC, Timpson NJ, Najjar SS, Stringham HM (2008). Newly identified loci that influence lipid concentrations and risk of coronary artery disease. Nat Genet.

[17] White H (1980). A heteroskedasticity-consistent covariance matrix estimator and a direct test for heteroskedasticity. Econometrica.

[18] Lt H, Bentler PM (1999). Cutoff criteria for fit indexes in covariance structure analysis: conventional criteria versus new alternatives. Struct Equ Model.

[19] Tucker L, Lewis C (1973). A reliability coefficient for maximum likelihood factor analysis. Psychometrika.

[20] Bentler PM (1990). Comparative fit indexes in structural models. Psychol Bull.

[21] Johansen CT, Kathiresan S, Hegele RA (2011). Genetic determinants of plasma triglycerides. J Lipid Res.

[22] Latsuzbaia A, Jaddoe VW, Hofman A, Franco OH, Felix JF (2016). Associations of genetic variants for adult lipid levels with lipid levels in children. The generation R study. J Lipid Res.

[23] Chasman DI, Pare G, Mora S, Hopewell JC, Peloso G, Clarke R, Cupples LA, Hamsten A, Kathiresan S, Mälarstig A (2009). Forty-three loci associated with plasma lipoprotein size, concentration, and cholesterol content in genome-wide analysis. PLoS Genet.

